# Refined control of CRISPR-Cas9 gene editing in *Clostridium sporogenes*: the creation of recombinant strains for therapeutic applications

**DOI:** 10.3389/fimmu.2023.1241632

**Published:** 2023-10-05

**Authors:** Aleksandra M. Kubiak, Luuk Claessen, Yanchao Zhang, Khashayarsha Khazaie, Tom S. Bailey

**Affiliations:** ^1^ Exomnis Biotech BV, Maastricht, Netherlands; ^2^ The M-Lab, Department of Precision Medicine, GROW - School for Oncology and Reproduction, Maastricht University, Maastricht, Netherlands; ^3^ Department of Immunology and Cancer Biology, Mayo Clinic, Phoenix, AZ, United States

**Keywords:** CRISPR-Cas9, *Clostridium sporogenes*, secretion, cancer, cytokines, immunotherapy

## Abstract

Despite considerable clinical success, the potential of cancer immunotherapy is restricted by a lack of tumour-targeting strategies. Treatment requires systemic delivery of cytokines or antibodies at high levels to achieve clinically effective doses at malignant sites. This is exacerbated by poor penetration of tumour tissue by therapeutic antibodies. High-grade immune-related adverse events (irAEs) occur in a significant number of patients (5-15%, cancer- and therapeutic-dependent) that can lead to lifelong issues and can exclude from treatment patients with pre-existing autoimmune diseases. Tumour-homing bacteria, genetically engineered to produce therapeutics, is one of the approaches that seeks to mitigate these drawbacks. The ability of *Clostridium sporogenes* to form spores that are unable to germinate in the presence of oxygen (typical of healthy tissue) offers a unique advantage over other vectors. However, the limited utility of existing gene editing tools hinders the development of therapeutic strains. To overcome the limitations of previous systems, expression of the Cas9 protein and the gRNA was controlled using tetracycline inducible promoters. Furthermore, the components of the system were divided across two plasmids, improving the efficiency of cloning and conjugation. Genome integrated therapeutic genes were assayed biochemically and in cell-based functional assays. The potency of these strains was further improved through rationally-conceived gene knock-outs. The new system was validated by demonstrating the efficient addition and deletion of large sequences from the genome. This included the creation of recombinant strains expressing two pro-inflammatory cytokines, interleukin-2 (IL-2) and granulocyte macrophage-colony stimulating factor (GM-CSF), and a pro-drug converting enzyme (PCE). A comparative, temporal *in vitro* analysis of the integrant strains and their plasmid-based equivalents revealed a substantial reduction of cytokine activity in chromosome-based constructs. To compensate for this loss, a 7.6 kb operon of proteolytic genes was deleted from the genome. The resultant knock-out strains showed an 8- to 10-fold increase in cytokine activity compared to parental strains.

## 1 Introduction

Immunotherapy has revolutionised modern clinical oncology. The concept of exploiting natural anti-microbial processes to steer the immune response against malignant tissue was exploited over a century ago by William Coley and led to attempts to enhance natural defences against cancer with immunogenised tumour cell vaccines (1). Earlier attempts with cytokine expression (2) and viral modification of tumour cells (3) produced promising results in animal models and led to clinical trials that are still ongoing (4, 5). A deeper understanding of novel immune system approaches for removing breaks through an immune checkpoint blockade rather than by stimulating immunity has introduced a new era of hope and expectation in cancer therapy (6–9). Nevertheless, major obstacles, including immune adverse effects and accelerated disease in response to therapy (10), remain to be understood and overcome. The search for complimentary approaches has revitalized attempts for targeting the tumour microenvironment through pharmacologic or immunologic strategies (3, 4, 11, 12). Tumour lytic viruses (13, 14) [reviewed in detail by Syyam et al. (15)] and bacteria (16) [reviewed in detail by Hu et al. (12)] have been at the forefront of the latter strategy in clinical attempts to hinder advanced cancers.

The observation that bacteria can colonise and persist in cancerous tumours is a long-standing one. Reports as early as 1813 observed regression of pre-existing tumours following gas gangrene infections, but more recent reports document the existence of complex communities of bacteria in tumours. This brings attention to several opportunities and raises further questions. In most cases, tumour-resident bacteria are opportunistic inhabitants rather than the initial cause of the cancer (17). The source of this “tumour microbiome” is not clear, but the gastrointestinal (GI) tract is an obvious candidate. There is evidence, both experimental and natural, of translocation from the GI tract (18, 19). Using modern RNA and DNA sequencing techniques, the profile of this community can be determined (20). This profile could be exploited to predict cancer prognosis and treatment response (21) or to improve treatment strategies (22). While a range of bacterial species, both obligately and facultatively anaerobic, have been reported to occur in tumours, the presence of *Clostridium* species is a recurring theme, which neatly links back to similar reports 200 years ago (23). A major advance in the use of bacteria for targeting of tumours was the creation of an attenuated strain of *Clostridium novyi*, an obligately anaerobic clostridia (24). *Clostridium novyi*-NT entered clinical trials (16) after extensive animal modelling in mice, rats, and dogs, showing promising results particularly in large animal clinical trials (25). Other examples include *Salmonella* vaccines (26, 27), which have been attenuated through genetic modifications (28) or self-destructive derivatives to overcome potential safety concerns (29). More recent attempts involve the use of viruses or bacteria for the intratumoural delivery of cargo, including tumour antigens (30).

Whether viral or bacterial, the challenges to using these vectors for intratumoural delivery of therapeutics are common. Appropriate selection of therapeutic agents aside, clinical efficacy will depend on the potency and dose of the agent at the site of disease. Since its inception, numerous innovations have yielded vast gains in the specificity and potency of immunotherapeutic agents, at least preclinically. Amino acid substitutions and fusion to receptor or antibody domains have enabled improvements in both the target affinity and half-life of the agents (31). Whether or not to increase the dose of the therapeutic is a decision that lies in the hands of the synthetic biologist and is something that draws on vast collective multidisciplinary knowledge. In the case of bacterial vectors, a rational approach is to optimise the steps between the DNA of the recombinant gene and the malignant target: transcription, translation, and secretion. Screening of gene promoters may be the simplest approach to increase transcription, and this has yielded impressive results in *Bacteroides* species (32). Increasing mRNA stability could also enhance expression, but it remains poorly characterised (33). Secretion, at least via the Sec pathway, has been studied extensively in several bacteria, including the model organism *Bacillus subtilis* (34–39). Equivalent systematic studies are yet to be conducted for *Clostridium* species.

The ability to precisely edit the genome brings significant benefits to therapeutic strain development. Through deletion of specific genes or modification of their expression, the phenotype of the host can be adjusted to suit any given application. Genome editing also enables containment of genetic elements, while plasmid-based expression cassettes introduce the risk of horizontal gene transfer, both in the environment (40) and within mammalian hosts (41, 42). Integration of therapeutic genes averts these risks and reduces the rate of mutation (43–45). In the last 10 years, numerous methods for gene editing in *Clostridium* species have been reported, the majority of which are based on CRISPR-Cas systems (46–50). While these illustrate that considerable progress has been made in this area, existing systems are hindered by low plasmid transfer efficiency and a lack of potency.

This paper reports the development of *C. sporogenes*, an obligate anaerobic, spore-forming bacteria, as a therapeutic delivery vector. Building on previous work (51–53), the aim of this study was to overcome the obstacles that have prevented this approach from becoming a viable clinical product. Specifically, our aim was to develop a synthetic strain that can produce functional product at therapeutic levels from a chromosome-based heterologous expression cassette. In pursuit of these objectives, a highly efficient and quick two-plasmid CRISPR-Cas9 system that enables deletion and insertion of large sequences (≥7 kb) was developed. Using the improved method, recombinant strains producing either cytokines or prodrug-converting enzyme (PCE) were created and validated. In addition, modification of the host’s phenotype through the targeted deletion of proteolytic genes resulted in a 7-fold increase of heterologous product.

## 2 Materials and methods

### 2.1 Bacterial strains, growth conditions and cell lines

Details of all bacterial strains used in this study are listed in **Table S1**. *C. sporogenes*-NT, a non-haemolytic strain of *C. sporogenes* NCIMB 10696 (henceforth referred to as WT), was created previously by deleting the putative Streptolysin S (*SLS*) operon (51). Two *Escherichia coli* strains (10-beta and S17-1) were used as the expression and conjugative donor strains, respectively.

Growth of *C. sporogenes* strains was carried out under anaerobic conditions in an anaerobic cabinet (model MG1000 Mark II, Don Whitley Scientific Ltd.; 80% N_2_, 10% CO_2_, 10% H_2_) at 37°C. *C. sporogenes* was grown in a bovine-free version of TY media, labelled “Peptone Yeast Thioglycolate” (PYT), supplemented with D-cycloserine (Cs, 250 μg/ml), thiamphenicol (Tm, 15 μg/ml), erythromycin (Erm, 30 μg/ml), and anhydrotetracycline (aTc, 96 ng/ml) where appropriate.

*E. coli* strains were grown aerobically at 37°C and 200 rpm in liquid LB medium or solid LB with 1.5% agar supplemented with Erm (300 μg/ml) or chloramphenicol (Cm, 25 μg/ml for solid media and 12.5 μg/ml for liquid media) or aTc (32 or 96 ng/mL).

Murine bone marrow FDCP-1 (“Factor-Dependent Continuous cell line, Paterson Laboratories”, ACC 368, DSMZ) and cytotoxic T-cells CTLL-2 cell lines (93042610, ECACC) were cultivated according to the manufacturer’s instructions.

### 2.2 Generation of plasmid-based cargo strains in *C. sporogenes*-NT

Details of all vectors and specific oligonucleotides used in this study are listed in **Tables S1** and **S2** of the **Supplementary Material**. For amplification of genomic or plasmid DNA, a high-fidelity polymerase was used according to the manufacturer’s protocol (Phusion Master Mix with HF buffer, M0531, New England Biolabs, NEB).

To construct plasmids harbouring three different cargos, FMN reductase - NfrA, murine interleukin 2 (mIL-2) and murine granulocyte-macrophage colony-stimulating factor (mGM-CSF), the DNA sequences were codon optimised according to the known codon usage preference of *C. sporogenes* and based on the reported sequence (UniProtKB: P39605, P04351, P01587). DNA fragments of desired cargo (NfrA, mIL-2 or mGM-CSF) and promoter/promoter-signal sequences were commercially synthesised (Integrated DNA Technologies, IDT Inc.) with sites recognized by type IIS restriction enzymes (**Table S3**).

The pGG212L vector was used as described before (53) to generate three constructs via Golden Gate Assembly (GGA), using *Bsa*I sites. Ligated plasmids were transformed by heat shock into *E. coli* 10-beta and plated on LB plates supplemented with Cm. Sequence-confirmed plasmids were either conjugated to *C. sporogenes*-NT or used as a donor DNA in the CRISPR-Cas9 integration stages. Resultant transconjugants have been confirmed by colony PCR using primers specified in **Table S2** and by Sanger sequencing (Azenta Life Sciences, formerly Genewiz, Inc.).

### 2.3 Creation of two-plasmid CRISPR-Cas9 editing plasmids

#### 2.3.1 Construction and conjugation of the tetracycline inducible Cas9 vector

**Figure 1:**
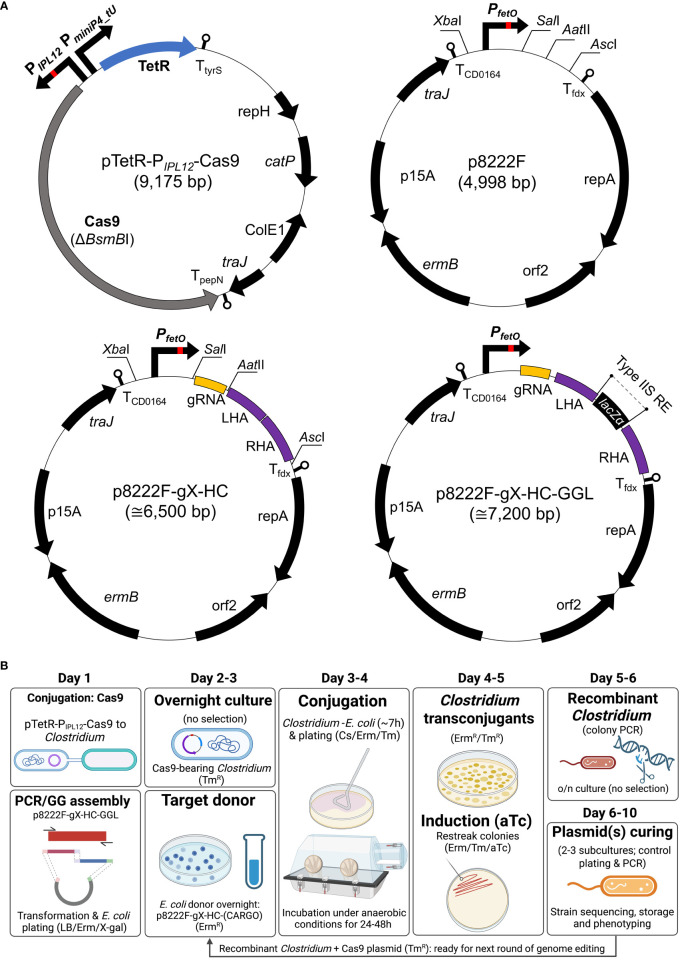
Schematic representation of the two-plasmid gene editing system. **(A)** pTetR-P*_IPL12_*-Cas9 carries the native SpCas9, modified to remove a *BsmB*I restriction site. The *tet* repressor gene (*tetR*) is expressed constitutively and binds to the tet operator (*tetO*) sequence of the synthetic P*_IPL12_* promoter in the presence of (anhydro)tetracycline. This is the basis for control of *cas9* expression. Both vectors utilised backbones from pMTL8000 vectors (54); gRNA, guide RNA consisting of spacer and scaffold sequences; LHA/RHA: left/right homology arm; *lacZα*, β-galactosidase counter-selection marker (for cargo cloning). **(B)** A representative workflow for gene editing using the CRISPR-Cas system. Conjugative transfer of pTetR-P*_IPL12_*-Cas9 into the host is conducted prior to and separately from that of p8222F-gX-HC. In parallel, a gRNA spacer sequence and the DNA (that is to be integrated) is cloned into p8222F-gX-HC. Following conjugative transfer of p8222F-gX-HC, viable colonies are restreaked to induction plates, which contain Erm, Tm, and aTc, and incubated for 24h. Cultures able to grow on induction plates are screened by PCR to detect genomic recombination. Plasmid loss is achieved by subculture in the absence of antibiotic selection. If Erm selection is maintained, pTetR-P*_IPL12_*-Cas9 is retained by the host and additional rounds of gene editing can be conducted. **(B)** created with BioRender.com.

The aTc inducible Cas9 vector pTetR-P*_IPL12_*-Cas9 is schematically illustrated in **Figure 1A**. Using pMTL83151 as template (54), a backbone fragment was created by polymerase chain reaction (PCR) using forward and reverse primers complementary to sequences upstream of pCB102 and downstream of *traJ*. The codon optimised *tetR* gene and divergent promoter sequences, consisting of P*IPL12* and P*miniP4_tU* (53), were designed in silico as a fusion sequence with flanking *Bsa*I restriction sites and commercially synthesised (IDT Inc.). Using two primer pairs, the *cas9* gene was amplified from *Streptococcus pyogenes* M1 genomic DNA as two overlapping fragments, each containing 5’ *Bsa*I restriction sites. In order to remove a *BsmB*I restriction site present in the native *cas9* coding sequence, a synonymous codon change (CGT to AGA) was introduced into the reverse primer of the 5’ cas9 fragment. Two new terminators—derived from the *Lactococcus lactis pepN* gene and the *B. subtilis tyrS* gene—were incorporated downstream of *cas9* and *tetR*, respectively. The terminators were split between adjacent plasmid fragments and incorporated as 5’ extensions to primers. The resulting four fragments were ligated using the Golden Gate Assembly protocol (NEB) to form the final plasmid. The inducible pTetR-P*_IPL12_*-Cas9 vector was transferred by conjugation to each variant of *C. sporogenes*.

#### 2.3.2 Construction of knock-out vectors

To construct the non-targeting vector, p8222F, an inducible ferredoxin promoter (P*fetO*) was designed to include *Xba*I at the 5’-end and *Sal*I-*Aat*II-*Asc*I cloning site at the 3’-end, and it was synthesized by IDT (**Figure 1A**; **Table S3**). For final construction, the inducible promoter was inserted into the pMTL82221 vector chassis via *Xba*I and *Asc*I restriction sites (54).

gRNA protospacer sequences were selected using the CRISPOR web tool (55). Cloneable gRNA fragments were created using a conserved reverse primer (corresponding to the 3’ of the gRNA handle and terminator) and a forward primer containing the spacer sequence at the 5’ and the beginning of the gRNA handle at the 3’. Complementary sequences between these primers enabled the creation of a product in a template-free “primer dimer” reaction. A *Sal*I and *Aat*II restriction site were incorporated up- and downstream of the gRNA product through 5’ primer sequences. To generate editing templates (repair cassette), sequences of approximately 750 bp long upstream and downstream of the chosen location were amplified from the *C. sporogenes* NCIMB 10696 genomic DNA, incorporating type IIS restriction sites. Fragments designated as left and right homology arms (LHA/RHA) were assembled using Golden Gate method. To demonstrate and exemplify the conjugation efficiency and tight regulation of tetracycline induction, a p8222F-sg7 vector was constructed, containing only *SLS*-targeting sgRNA module, without repair cassette.

#### 2.3.3 Construction of Golden Gate compatible knock-in vectors

Two Golden Gate compatible knock-in vectors (p8222F-g7-SLS-GGL and p8222F-g2-PyrE-GGL) were constructed, targeting the *SLS* operon and *pyrE* gene, respectively (**Figure 1A**; **Supplementary Figure S1**). The Golden Gate-adapted *lacZa* module (56) was amplified and ligated between the repair cassette flanking *SLS* operon or *pyrE* gene, respectively.

To generate CRISPR-Cas9 editing plasmids with the desired cargo, p8222F-g7-SLS-GGL or p8222F-g2-PyrE-GGL were used in a GGA reaction alongside previously constructed pGG212 harbouring NfrA, mIL-2, or mGM-CSF, respectively, with *BsmBI* and T4 DNA ligase. Ligated plasmids were transformed by heat shock into *E. coli* 10-beta and plated on LB plates supplemented with Erm. Resultant colonies were PCR-screened for correct assembly and subsequent plasmid samples were confirmed by Sanger sequencing.

### 2.4 Generation of *C. sporogenes* knock-out and integrant strains

Constructed knock-out and knock-in vectors were heat shock transformed into *E. coli* S17-1 and conjugated into *C. sporogenes* strain harbouring pTetR-P*_IPL12_*-Cas9 plasmid. Conjugation was carried out as previously described (54) with some additional considerations. A critical ratio of 1: 5 (donor to recipient) was followed. Prior to conjugation, the recipient strain was heat shocked for 2 minutes at 55°C (57). Donor-recipient mixture was plated on PYT media supplemented with both Erm and Tm. Transconjugant colonies were subsequently re-streaked on PYT plates with the addition of aTc inducer. The procedure is schematically illustrated in **Figure 1B**.

Resultant knock-out or integrant strains were screened by colony PCR using primers that flank the genome locus (**Table S2**). The editing vector and pTetR-P*_IPL12_*-Cas9 vector were removed from knock-out mutants by subculturing in non-selective liquid cultures (24-48h) followed by patch plating to selective and non-selective plates to observe loss of antibiotic resistance. Plasmid loss was further confirmed by PCR specific for the plasmid-based *traJ* gene (data not shown). Resultant positive clones were sent for sequencing. In the event where resultant recombinant strains were subjected to another round of integration with a different editing template, the pTetR-P*_IPL12_*-Cas9 vector was maintained.

#### 2.4.1 Measurement of conjugation efficiency

To calculate conjugation efficiency (CE), donor-recipient spot mixtures were incubated for 7 hours on non-selective plates before being resuspended in 0.5 ml PBS, serially diluted, and spread on selection plates containing Cs and Erm. When using *C. sporogenes* bearing the pTetR-P*_IPL12_*-Cas9 as the recipient, Tm was added to plates to maintain the Cas9 plasmid in transconjugants. Additionally, the conjugation mixtures were also plated on relevant selection plates with the addition of aTc as the inducer (96 ng/ml). Approximate conjugation efficiencies were then calculated as transconjugant colony forming unit (CFU) per total *C. sporogenes* recipient CFU.

### 2.5 Phenotype analysis of *C. sporogenes* knock-out and integrant strains

#### 2.5.1 Growth and endospore formation for integrant strains

The ability of spores of *C. sporogenes* integrants to return to vegetative growth was evaluated by measuring the change in OD_600_ over a period of 24 hours. Overnight cultures were inoculated into a fresh PYT media (1:100) and incubated at 37°C under anaerobic conditions. Additionally, uracil auxotrophy for *C. sporogenes* strains lacking the *pyrE* gene was evaluated by plate assay using agar plates with a chemically defined composition of media and supplemented with uracil (20 μg/ml) where appropriate. Endospore formation titres for *C. sporogenes* integrant strains were evaluated at 120h time point. Briefly, 1 ml samples were treated for 20 min at 80 ˚C to heat-kill vegetative cells and plated in serial dilutions (from 10^-1^ to 10^-7^ in sterile and anaerobically reduced PBS) on PYT plates supplemented with Cs. After 24-48h anaerobic incubation, colonies from agar plates were enumerated and spore titre calculated as CFU/ml. A *spo0A* mutant (CspWT-Δ*spo0A*), in which the master regulator of sporulation has been deleted, was used as a negative control for colony formation after heat treatment.

#### 2.5.2 Detection of recombinant mIL-2 and murine GM-CSF

To quantify the levels of cytokines secreted from either plasmid-based or integrant strains of *C. sporogenes*, supernatant samples were obtained from three different growth time points (5h, 7h and 10h) during bacterial growth. Subcultures of mIL-2 or mGM-CSF-expressing *C. sporogenes* were centrifuged (10,000 *x g*, 10 min), filtered (0.22 μm syringe filter), and the sterile supernatant retained.

The levels of mIL-2 and mGM-CSF were determined by cytokine-specific enzyme-linked immunosorbent assay (ELISA) in accordance with the manufacturer’s instructions (88-7334; 88-7334, Invitrogen). The results were recorded in a microplate reader (iD3, SpectraMax) and calculated based on recombinant mIL-2 and mGM-CSF standards and normalised to OD_600 _= 1 to account for differences in growth rates.

The biological activities of the secreted cytokines were determined in a lymphocyte proliferation assay using the CTLL-2 or FDCP-1 T cell line. Supernatant-stimulated proliferation was measured using either an MTT assay (for CTLL-2) according to published methods (58) or AlamarBlue (for FDCP-1). The standard curve was prepared using purified recombinant mIL-2 or mGM-CSF (212-12 and 315-03, Peprotech). For the MTT assay, absorbance was recorded at 470 nm. AlamarBlue assay results were recorded using the fluorometric read-out at 560/590 nm excitation/emission, in a multimode microplate reader (iD3, SpectraMax).

#### 2.5.3 Menadione reductase assay

To compare the activity of NfrA nitroreductase expressed in plasmid-based and integrant strains, menadione reductase activity was determined by the method of Knox et al. (59). To do this, 7-hour samples of 2 ml cultures were harvested by centrifugation. Cell pellets were subjected to BugBuster cell lysate preparation according to manufacturer’s recommendations. The assay was performed in 1 ml plastic cuvettes and a spectrophotometer set for 37°C. The increase of absorbance at 550 nm for 1 min was recorded and the rate divided by the volume of lysate (in ml). The extinction coefficient (E^mM^) of cytochrome *c* was used at 14.79. To normalise the units of activity per weight of protein, a bicinchoninic acid assay (BCA) was performed in accordance with manufacturer’s guidelines (23225, Thermo Scientific). The final menadione NfrA activity was expressed in units per mg (U/mg).

### 2.6 Gelatin zymography and quantitative analysis of proteolytic activity

To detect and compare the levels of proteases in wild-type or recombinant *C. sporogenes* strains, gelatin zymography was performed using 10% Novex^®^ Zymogram Gel according to manufacturer’s instructions (MAN0005885, Novex/Invitrogen). To do this, samples from cultures of *C. sporogenes*-NT-Δ*pyrE* and *C. sporogenes*-NT-Δ*pyrE*ΔPR1 were taken at four different time points (5h, 7h,10h, and 12h), centrifuged (10,000 *x g*, 10 min) and filter sterilised (0.22 μm syringe filter). Sterile supernatants, normalised according to cell density (OD_600 _= 1.2), were mixed with Tris-Glycine SDS Sample Buffer (2x), of which 20 μl was loaded under non-reducing conditions into precast gelatin zymography gels. *Clostridium butyricum* DSM 10702 supernatant sample, obtained from 10-hour growth, was used as a control. The zymograph was run at 125 V for 90 minutes.

To quantify the total protease activity in culture supernatants, a colorimetric protease assay was performed in 96-well plates, according to manufacturer’s recommendations (23263, Thermo Scientific™ Pierce™ Colorimetric Protease Assay Kit). The spectrophotometric measurement was recorded using a microplate reader (iD3, SpectraMax). Comparative protease activities were calculated and expressed in ng/ml using trypsin standard curve as a reference.

### 2.7 Biological replicates and statistical analyses

All data presented in this manuscript represent the results of at least two independent experiments. Statistical evaluations were performed with GraphPad Prism 9 software (San Diego, CA, United States). For the ELISA, cell proliferation and protease assays, and for the conjugation efficiencies, data were analysed using unpaired t-test to compare strain variants analysed at different time points or conditions. Values of P<0.05, P<0.01, P<0.001 were considered significant (∗), highly significant (∗∗), or extremely significant (∗∗∗) respectively. Data represent means ± standard deviation (SD).

## 3 Results

### 3.1 Two-plasmid, inducible CRISPR-Cas9 system improves editing efficiency in *C. sporogenes*

In the present study, we have constructed a genome editing system based on two plasmids: a *cas9*-expression plasmid and a second plasmid bearing the gRNA and repair cassette. Expression of the *cas9* gene is controlled using a tetracycline (*tet*) inducible promoter (60). A schematic representation of the plasmid is shown in **Figure 1A**. Prior to the creation of this plasmid, gene expression control using the *tet* repressor and operator was confirmed in *E. coli* using the *gusA* (β-glucuronidase, GUS) reporter (**Supplementary Figure S2**).

In order to test the new system, a series of plasmids, designed to knock-out sequences of the *C. sporogenes* genome, were created. Using the labelling scheme p8222F-gX-HC, these plasmids contain the gRNA (“X”) under the control of the *tet* inducible P*fdx* promoter (P*fetO*) and the target-specific homology cassette (“HC”). Four genome loci were targeted: *pyrE* (609 bp), *spo0A* (1.2 kb), *spoIIE* (2.4 kb), and the *SLS* operon (8.64 kb).

Prior to mutagenesis, the pTetR-P*_IPL12_*-Cas9 plasmid was conjugated to the *C. sporogenes* strain of interest (*C. sporogenes* WT or *C. sporogenes*-NT). These *cas9*-bearing cultures were then used as conjugation recipients of the second plasmid. Following induction, deletion of the target sequence was detected in all clones screened by flank PCR, indicating 100% efficiency (**Supplementary Figures S3A, B**).

Poor conjugation efficiency has the potential to distort reported genome editing efficiency. To demonstrate that perceived high genome editing efficiency is not an artefact of low transconjugant yield, we sought to demonstrate the conjugation performance of this system in the presence and absence of inducer. This was achieved by normalising transconjugant CFUs based on recipient titre, referred to henceforth as conjugation efficiency (CE). In addition, we evaluated the effect of cargo size on p8222F CE. Plasmids bearing the repair cassette and/or sgRNA were targeted to the *SLS* locus. Following conjugation, CE was determined both for empty *C. sporogenes* and for *C. sporogenes* bearing pTetR-P*_IPL12_*-Cas9. In order to assess the effect of Cas9 and gRNA expression on conjugation in the absence of a repair cassette, a plasmid bearing the *SLS* gRNA, but without a repair cassette, was constructed (p8222F-sg7).

**Figure 2:**
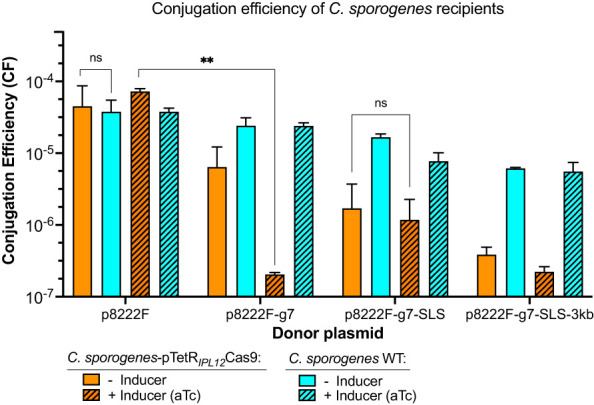
The effect of the size and the composition of four different vectors on the conjugation efficiencies of two recipients: empty *C. sporogenes* WT and strain bearing the inducible Cas9-plasmid. p8222F (4,998 bp): plasmid with only P*_fetO_* promoter; p8222F-g7 (5,128 bp): plasmid with addition of sgRNA targeting *SLS* operon; p8222F-g7-SLS (6,567 bp): plasmid targeting *SLS* operon with repair cassette; p8222F-g7-SLS-3kb (9,698 bp): plasmid targeting *SLS* operon with repair cassette and 3 kb λDNA cargo. The transconjugants were counted on selection plates: Erm/Cs for *C. sporogenes* WT (in turquoise) and Erm/Cs/Tm for C. *sporogenes*-pTetR-P*_IPL12_*-Cas9 (in orange) with and without inducer (aTc, 96 ng/ml). Approximate conjugation efficiencies were calculated as obtained transconjugant CFU/total *C*. *sporogenes* recipient CFU. Each bar represents the mean and standard deviation of data collected from two experiments performed using biological duplicates. Statistical analysis was performed using unpaired t-test, ns – not significant, ** P<0.01.

Conjugation of p8222F, in which gRNA and repair cassette are absent, showed similar efficiency in both recipients, indicating the presence of pTetR-P*_IPL12_*-Cas9 does not affect conjugation (**Figure 2**). The addition of inducer did not influence p8222F CE for either recipient. The addition of gRNA (p8222F-g7) caused a non-significant decrease of CE in *C. sporogenes* pTetR-P*_IPL12_*-Cas9, which was reduced further in the presence of inducer (P=0.002). This was not observed when empty *C. sporogenes* was used as the recipient. The addition of the repair cassette (p8222F-g7-SLS) appears to “rescue” CE in *C. sporogenes* pTetR-P*_IPL12_*-Cas9, with comparable efficiencies in the presence and absence of inducer. The inclusion of 3 kb of “cargo” (p8222F-g7-SLS-3kb) reduced CE, with or without inducer, in *C. sporogenes* pTetR-P*_IPL12_*-Cas9 but the difference was not statistically significant.

### 3.2 Golden Gate-compatible vectors streamline cargo cloning

In order to streamline cargo cloning, plasmids targeted to two genome loci were engineered to contain golden gate cloning sites between the homology arms (56), referred to as p8222F-g2-PyrE-GGL and p8222F-g7-SLS-GGL. These plasmids target the *pyrE* locus and *SLS* locus, respectively. Using GGA, NfrA, mIL-2 and mGM-CSF were cloned into p8222F-g7-SLS-GGL. Based on the results of previous work, the native promoter of the ferredoxin (P*fdx*) gene was selected to ensure strong expression of the recombinant proteins (53), and the native secretion peptide of *nprM3* to promote efficient secretion (51). Using *C. sporogenes* WT and *C. sporogenes*-Δ*pyrE* as hosts, knock-in mutants of NfrA, mIL-2 and mGM-CSF were created, resulting in *C. sporogenes*-NT and *C. sporogenes*-NT-Δ*pyrE* versions of each recombinant strain (**Supplementary Figure S4A**). Deletion of the *pyrE* gene has been shown to cause uracil auxotrophy (52), and could serve as a method for biocontainment of genetically engineered *C. sporogenes* when used in clinical applications. Although we have only utilised the *SLS*-targeting plasmid in this study, the creation of the p8222F-g2-PyrE-GGL plasmid enables integration at the *pyrE* locus in a second round of genome editing, using *C. sporogenes*-NT as the host. Other loci can be targeted using the same rational.

In order to test the ability of the CRISPR-Cas9 system to integrate large DNA fragments, two arbitrary fragments of lambda DNA were created and cloned between the homology arms. The largest cloned fragment was 5 kb, resulting in a total plasmid size of 11.7 kb. Larger fragments were not tested as the efficiency of both *E. coli* transformation and *C. sporogenes* conjugation is low for plasmids above 12 kb. Based on total colonies screened, integration efficiencies were 91.66-100% for cargo sizes ranging from 835 bp to 5,256 bp (**Supplementary Figure S4B**).

### 3.3 Chromosome-based intracellular and secreted products are biologically active

**Figure 3:**
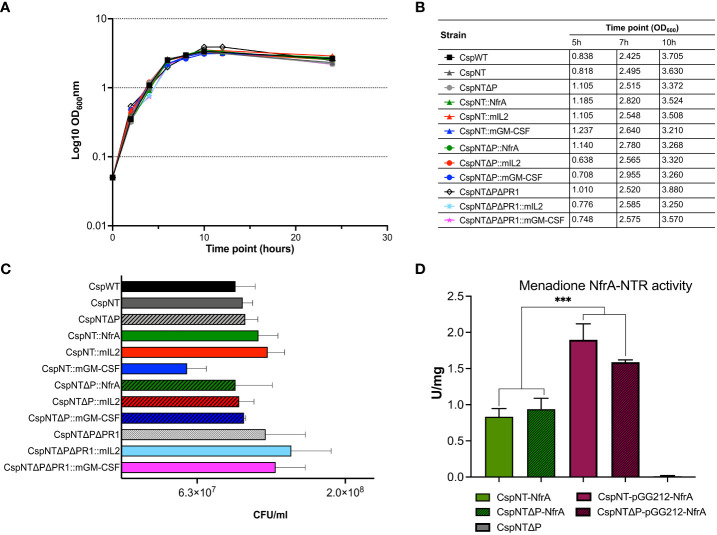
Characterisation of growth, sporulation, and enzymatic activity assay in *C*. *sporogenes* strains. **(A)** Growth of *Clostridium* strains was measured as a direct increase in absorbance at 600 nm throughout the course of 24-hour bacterial incubation in bovine free medium (PYT). The symbols represent the average of three independent experiments, and error bars indicate the standard errors of the means. **(B)** The summary of OD_600_ measurements recorded at the times of pre-determined experimental time point sample collections (5h, 7h and 10h). **(C)** Spore titres after 120h incubation in PYT broth. Samples of heat-treated cultures (80°C, 20 min) were plated in serial dilution on agar plates and enumerated following 24-48h incubation. Bars represent the number of CFU (colony forming unit) per ml of culture. The data represent the average of three independent experiments and error bars indicate the standard error of the mean. The sporulation-deficient *Clostridium sporogenes-*NT-Δ*spo0A* mutant was used as a negative control to rule out experimental error. The detection limit for colony counts was 50 CFU/ml. **(D)** Menadione reductase activity assays on *C*. *sporogenes* cell lysates containing either plasmid-based or chromosome-integrated *nfrA* gene. Results show specific menadione reductase activities of *C*. *sporogenes* NfrA-expressing strains soluble fractions obtained from 7h time points during the growth of bacterial cultures. The *C*. *sporogenes*-NT-Δ*pyrE* was used as background control. Activities were normalised to total protein concentrations, determined using BCA assay. Activities are expressed in units per mg. The data represent the average of three independent experiments and error bars indicate the standard errors of the means. Statistical analysis was performed using unpaired t-test, *** P<0.001.

Clones of six recombinant strains were taken forwards for biochemical and functional validation. The recombinant strains were generated in two different backgrounds: Δ*SLS* and Δ*SLS*Δ*pyrE*. Growth on defined media agar plates with or without uracil was tested (**Supplementary Figure S5**). Recombinant strains generated in the Δ*SLS*Δ*pyrE* background were not able to grow in the absence of uracil, indicating they are auxotrophic. In contrast, strains generated in the Δ*SLS* background did not require uracil supplementation. To further characterise the new strains, growth over 24 hours in rich media and ability to form spores over 120 hours was tested. Growth rate and sporulation of both the recombinant strains and the knock-out strains were somewhat similar to those of *C. sporogenes* WT (**Figures 3A–C**). The results of these assays indicate that addition of heterologous expression cassettes does not have a significant effect in these processes under the conditions tested.

The nitroreductase enzyme NfrA is expressed intracellularly and can be quantified using the menadione assay, as reported previously (52). Recombinant strains expressing NfrA from a plasmid or the chromosome were generated in the Δ*SLS* and Δ*SLS*Δ*pyrE* backgrounds. Host genotype did not affect nitroreductase activity, but plasmid-based strains produced significantly more activity than chromosome-based equivalents (P<0.0004) (**Figure 3D**).

**Figure 4:**
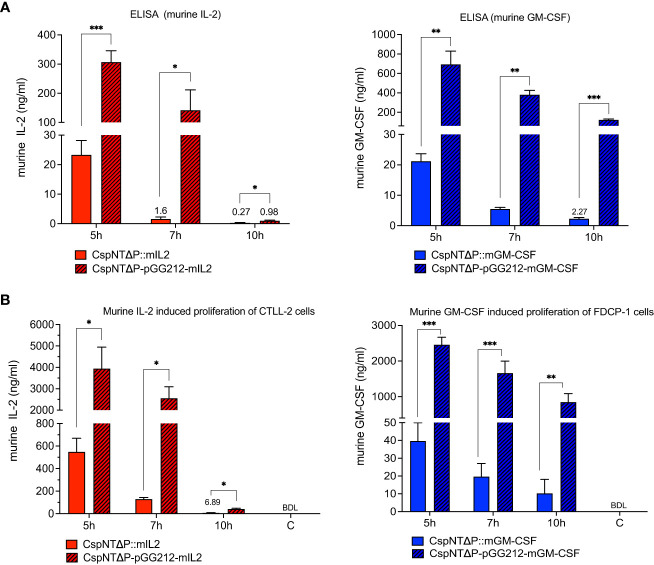
Validation of *C*. *sporogenes*-NT-Δ*pyrE* strains harbouring either plasmid-based or integrated murine cytokines (mIL-2 and mGM-CSF) at the *SLS* locus in the quantitative and functional assays. **(A)** Results of commercial ELISA tests indicating the quantities of mIL-2 and mGM-CSF cytokines present in the supernatants of cytokine bearing-*C. sporogenes* strains at 5-,7-, and 10-hour growth in PYT media. **(B)** Results of MTT and AlamarBlue functional assays following the incubation of cytokine specific T-cells in the presence of culture supernatants of *C*. *sporogenes* strains. Recombinant murine IL2 and GM-CSF were used to prepare mIL-2 and mGM-CSF standard curves., (C) denotes *C*. *sporogenes*-NT-Δ*pyrE* control, (BDL) - below detectable levels. Statistical analysis was performed using unpaired t-test, * P<0.05, ** P<0.01, *** P<0.001.

Levels of secreted mIL-2 and mGM-CSF in culture supernatants after 5, 7, and 10 hours of growth were quantified by ELISA. Supernatants from cultures of equivalent plasmid-based constructs were taken at the same time points for comparison. Cell density was measured for each sample at each time point (**Figure 3B**), and calculated cytokine levels were normalised to OD_600 _= 1. In strains of both cytokines, maximum product levels occurred after 5 hours of growth, with sequential reductions at 7 and 10 hours (**Figure 4A**). Product levels were substantially (more than 10-fold) higher from plasmid-based expression cassettes at all time points compared to the same cassette integrated into the chromosome.

Cytokine function was evaluated in cell proliferation assays using the CTLL-2 and FDCP-1 cell lines for mIL-2 and GM-CSF, respectively (**Figure 4B**). In line with the ELISA results, peak levels of mIL-2 (3.9 µg/ml) and mGM-CSF (2.5 µg/ml) occurred in cultures of plasmid-based strains after 5 hours of growth. At the same time point, chromosome-based constructs produced substantially less product (0.5 µg/ml and 0.04 µg/ml, respectively). For the plasmid-based strain, levels of mIL-2 were reduced at 7 hours (-35%) and almost undetectable at 10 hours (-98%). In contrast, levels of mGM-CSF from the plasmid-based strain remained relatively high at all time points, reducing sequentially by 32% and 50% at 7 and 10 hours, respectively.

### 3.4 Deletion of the 7 kb nprM operon increases the yield of secreted cytokines

**Figure 5:**
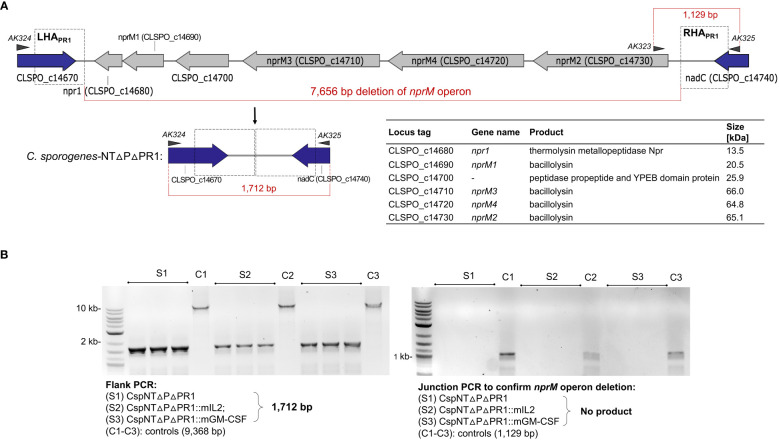
Schematic representation of *nprM* proteolytic operon knock-out in *C*. *sporogenes* mediated by inducible two-plasmid CRISPR-Cas9 system. **(A)** Deletion of six proteolytic genes has been exemplified with *C*. *sporogenes*-NT-Δ*pyrE* strain and subsequently repeated using two cytokine-bearing integrant strains. Triangles represent indicative alignment of screening primers as presented in **Table S2**. **(B)** Gel electrophoresis showing PCR screens of all three proteolytic knock-out strains conventionally labelled as ΔPR1, where S1: CspNTΔPΔPR1 (C. *sporogenes*-NT-Δ*pyrE*ΔPR1), S2: CspNTΔPΔPR1::mIL-2 (C. *sporogenes*-NT-Δ*pyrE*ΔPR1::mIL-2) and S3: CspNTΔPΔPR1::mGM-CSF (C. *sporogenes*-NT-Δ*pyrE*ΔPR1::mGM-CSF). Left image: Deletion of the operon detected in colonies subjected to a PCR reaction using primers flanking the deletion locus. Right image: Subsequent confirmation of proteolytic operon deletion in selected clones using internal (junction) and flanking primers. (L) denotes DNA marker, (C1-C3): equivalent *C*. *sporogenes*-NT-Δ*pyrE* controls.

Levels of secreted cytokine were significantly reduced in later phases of bacterial growth cultures. This led us to hypothesise that proteases secreted by the host bacteria were degrading the recombinant product. To test our hypothesis, we sought to investigate the impact that deleting proteolytic genes would have on the levels of secreted recombinant product. A bioinformatic screen of the *C. sporogenes* NCIMB 10696 genome revealed a 7.4 kb operon, annotated as six metallopeptidase genes (1,607,357 bp to 1,615,014 bp), henceforth referred to as the *nprM* operon. Using the same method as for *pyrE* and the *SLS* operon, the six genes were deleted using gRNA and repair cassettes targeted to the *nprM* operon (**Figure 5A**). This was conducted in *C. sporogenes-*NT-Δ*pyrE* and in the cognate mIL-2 and GM-CSF expressing strains. Deletion of the target operon (“ΔPR1”) was confirmed by PCR and Sanger sequencing (**Figure 5B**). The loss of these genes did not affect the host’s ability to grow or sporulate in rich media (**Figures 3A–C**).

**Figure 6:**
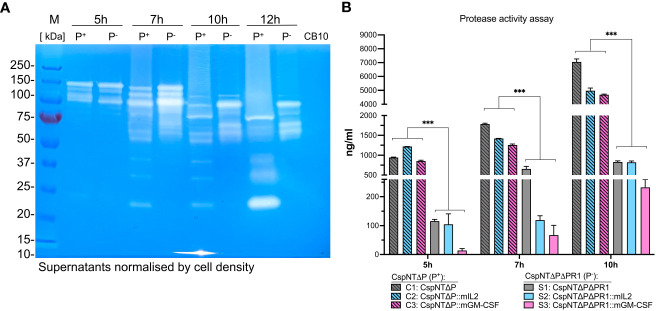
Analysis of proteolytic activity in *C*. *sporogenes* strains. **(A)** Qualitative analysis of proteolytic activity by gelatin zymography of the *C*. *sporogenes*-NT-Δ*pyrE* and *C*. *sporogenes*-NT-Δ*pyrE*ΔPR1 supernatants at four different time points (5h, 7h, 10h and 12h). In total, 20 μl of samples were loaded on a SDS polyacrylamide gel containing 0.05% of gelatin. After electrophoresis, proteins were renaturated in the gel and the proteolytic activity was revealed after an overnight incubation required for gelatin degradation. CB10 - C*. butyricum* control supernatant collected at 10h growth. Samples were normalised according to OD_600_ of 1.2 prior to loading. M: Pre-stained Protein Ladder PageRuler™ (Thermo Scientific™); P^+^: *C*. *sporogenes*-NT-Δ*pyrE* samples (PR1 positive); P^-^: *C*. *sporogenes*-NT-Δ*pyrE*ΔPR1 (PR1 negative); CB10 - C*. butyricum* control supernatant collected at 10h growth. **(B)** Quantitative analysis of proteolytic activities detected in *C*. *sporogenes* strains using Pierce™ colorimetric protease assay (Thermo Scientific™) and sample supernatants collected at three different time points (5h, 7h, and 10h). CspNTΔP (P^+^) and CspNTΔPΔPR1 (P^-^) – PR1-positive and PR1-negative backgrounds, respectively. Standard curve for the assay has been constructed using serially diluted TPCK trypsine stock solution. The absorbance measurement has been carried out at 450 nm. The data represent the average of two independent experiments, and error bars indicate the standard errors of the means. Statistical analysis was performed using unpaired t-test, *** P<0.001.

In order to visualise the effect of the *nprM* operon deletion on the levels of secreted proteins, a gelatin zymograph experiment was conducted (**Figure 6A**). For each time point (5, 7, 10 and 12-hour), sterile supernatants of CspNT-Δ*pyrE* and CspNT-Δ*pyrE*ΔPR1 were loaded, pairwise, onto the zymograph. Following electrophoresis, we observed a distinct difference at all time points between the two strains. In relative terms, the CspNT-Δ*pyrE* samples showed reduced quantities of high molecular weight proteases, but distinct lower molecular weight proteases that are absent in the CspNT-Δ*pyrE*ΔPR1 samples.

A colorimetric protease assay was conducted to quantify the impact of this deletion on extracellular proteolysis (**Figure 6B**). The Pierce Protease Assay detects proteolysis through the reaction of peptide fragments, created by hydrolysis of succinylated casein, with trinitrobenzene sulfonic acid (TNBSA). The resultant TNB-peptide adducts produce a yellow colour, which can be quantified by measuring absorbance at a 450 nm wavelength and comparison with a protease calibration curve. As for previous experiments, supernatants were collected at 5, 7, and 10h for three strains, mIL-2, GM-CSF, and parental, in two different backgrounds: Δ*pyrE* and Δ*pyrE*ΔPR1. This experimental design allowed us to compare proteolysis in different contexts and across time and to do multiple pairwise analyses of proteolytic activity. A statistically significant reduction in proteolysis was observed in Δ*pyrE*ΔPR1 strains compared to Δ*pyrE* strains at all time points (P<0.05). This held true for strains with and without recombinant products.

**Figure 7:**
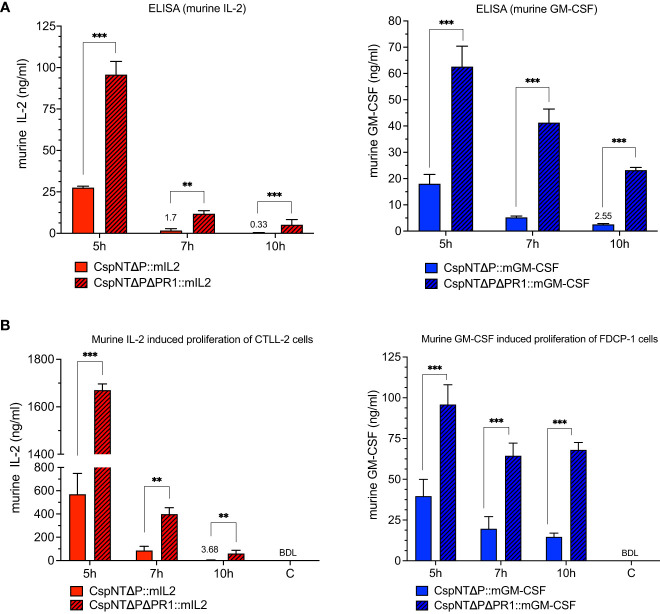
Validation and comparison of *C*. *sporogenes*-NT-Δ*pyrE* and *C*. *sporogenes*-NT-Δ*pyrE*ΔPR1 strains harbouring integrated murine cytokines (mIL-2 and mGM-CSF) in the quantitative and functional assays. **(A)** Results of commercial ELISA tests indicating the quantities of mIL-2 and mGM-CSF cytokines present in the supernatants of cytokine bearing-*C. sporogenes* strains at 5-,7-, and 10-hour growth in PYT media. **(B)** Results of MTT and AlamarBlue functional assays following the incubation of cytokine specific T-cells in the presence of culture supernatants of *C*. *sporogenes* strains. Recombinant murine IL-2 and GM-CSF were used to prepare mIL-2 and mGM-CSF standard curves. (C) denotes *C*. *sporogenes*-NT-Δ*pyrE*ΔPR1 control, (BDL) – below detectable levels. The data represent the average of three independent experiments and error bars indicate the standard errors of the means. Statistical analysis was performed using unpaired t-test, ** P<0.01, *** P<0.001.

Having established that the *nprM* knock-out strains showed reduced proteolysis, we sought to test our hypothesis that this phenotype would translate to increased recombinant product in culture supernatants. The ELISA and cell proliferation assays, detailed in **Figure 4**, were repeated using the CspNT-Δ*pyrE*ΔPR1 expressing mIL-2 and GM-CSF. Equivalent strains in the CspNT-Δ*pyrE* background (used in the previous experiment) were tested again in these assays. At all time points, recombinant product was increased in the CspNT-Δ*pyrE*ΔPR1 supernatants (**Figures 7A, B**). The difference was less pronounced at the earliest time point (5h), with increased ELISA/cell assay measurements of 244%/148% and 248%/142% for mIL-2 and GM-CSF, respectively. The difference in product levels was more pronounced at 7h but increased further to peak at 10h with increased ELISA/cell assay measurements of 1653%/1546% and 807%/363% for mIL-2 and GM-CSF, respectively. In all cases, the differences were statistically significant (P<0.05).

## 4 Discussion

The objectives of this study were two-fold: 1) to combine several improvements to CRISPR-Cas9 technology that result in the development of a cloning-friendly and efficient genetic tool for modification of *Clostridium* genomes and 2) to demonstrate the application of this tool in the development of oncologically relevant recombinant strains. To achieve this, a number of impediments had to be overcome. Although less of an issue in *C. sporogenes*, poor efficiency of transformation, by conjugation or electroporation, has been a major hindrance to strain development in related *Clostridium* species (61). Irrelevant of the transformation method, the inverse relationship between efficiency and plasmid size is difficult to overcome. To mitigate this, we chose to split the components of the CRISPR-Cas9 system across two plasmids. We constructed a 9.2 kb Cas9 expression plasmid (pTetR-P*_IPL12_*-Cas9) and a second, 6.6 kb editing plasmid (p8222F-gX-HC), containing the gRNA and repair cassette. The relatively small size of p8222F-gX-HC leaves significant headroom for the addition of sequences to be integrated before the overall size of the plasmid hinders cloning and conjugation. Transfer of DNA is considerably less efficient in other species of clostridia and is likely to necessitate further measures to facilitate transformation. Improvements have been achieved by selective DNA methylation to bypass host restriction-modification systems (62) and through heat-shock of the recipient prior to conjugation (57). The equivalent single plasmid system would require a plasmid of 11 kb or larger. An additional advantage of this approach is that multiple rounds of genome editing can be conducted following conjugation of the pTetR-P*_IPL12_*-Cas9 plasmid without the need to remove the Cas9 expression vector. To reduce the time and resources required to clone new sequences for integration, we incorporated the previously reported golden gate cloning site (56). This enables one-pot cloning reactions and blue-white colony screening. Using previously validated integration sites, such as those demonstrated in this paper, this system can be used to create libraries of integrant strains at a cost of time and resources that most academic labs could bear.

Obtaining transconjugants that bear both plasmids is significantly easier when Cas9 expression is repressed. Previous versions of this system relied on constitutive expression of gRNA or the *cas9* gene or both (63). By placing both Cas9 and gRNA expression under the control of inducible promoters, the inherent toxicity of Cas9, with or without gRNA, is alleviated. However, the reduced conjugation efficiency of all p8222F-based plasmids into a pTetR-P*_IPL12_*-Cas9-bearing host compared to plasmid-less host indicates that a degree of host toxicity remains, possibly due to incomplete repression of *cas9* transcription.

In this context, utilising inducible promoters for Cas9 and/or gRNA expression risks insufficient expression of these components, and a concomitant loss of potency. Ultimately this would result in a low frequency of mutant isolation, due to inadequate Cas9-mediated DNA cleavage and subsequent homology-directed repair. Assessment of a panel of *tet* inducible promoters in a GUS reporter assay indicated that expression from synthetic promoter *IPL12*-*miniP4*_tU was high in the presence of inducer, but indistinguishable from background in its absence (**Supplementary Figure S2**). Following conjugation and induction, our results indicate that all surviving colonies had recombined with the provided repair cassette to produce the desired mutant strains. This held true in tests with cargo sizes up to 5 kb. Combined with the data for CE and gene deletion, these results demonstrate that deletion of sequences up to 8.6 kb and addition of at least 5 kb can be achieved by a single user in a short amount of time without the need for high volume colony screens.

Chromosomal integration of foreign DNA offers several advantages compared to plasmid-based approaches. Transfer of extrachromosomal DNA between bacteria in multispecies communities is well documented, and maintenance of a plasmid typically requires the presence of antibiotic. The risk of introducing synthetic plasmids to other bacteria, within a patient or the environment, is likely to be a major hurdle for regulatory approval. Furthermore, plasmids are structurally and segregationally unstable, which can lead to unpredictable gene expression or inactivation of the gene. However, plasmid-based genes can achieve higher levels of expression than when they are chromosome-based.

We selected two oncologically relevant cytokines to assess the capability of the new gene editing system and to evaluate the impact of integration on biological activity. Interleukin-2, a potent T cell activator, is approved for the treatment of renal cell carcinoma and metastatic melanoma (64, 65), where high-dose regimens produce significant clinical responses (66) but are hindered by severe toxicity (67). Attempts to prevent toxicities using lower dosage regimens can cause a significant drop in therapeutic effect due to the dominant effect of immunosuppressive regulatory T cells (68). “Immune-related adverse effects” (irAEs) during immunotherapy are the result of the agent interfering with normal immune function in healthy tissues, and typically manifests as autoimmune-like symptoms. Agents that are hindered or, in some cases, unusable due to irAEs are ideal candidates for bacteria-mediated intratumoural delivery, which could, in theory, maximise the dose at the tumour site without requiring systemically high doses. As for IL-2, the influence of GM-CSF in anti-cancer immunity is dose-dependent. GM-CSF stimulates the production, maturation, and activation of innate immune cells, such as neutrophils, macrophages and dendritic cells. Thus, it is postulated that GM-CSF treatment could promote activation of the adaptive immune response indirectly by promoting tumour-reactive innate cells (69). In addition, strains expressing a pro-drug converting nitroreductase enzyme (NfrA), reported previously (52), were developed. NfrA is currently being investigated for its potential to convert PCE CP-506, entering phase 1/2 clinical trial (70). Due to the absence of a secretion peptide in the coding sequence of NfrA, this product remains inside the host cell. Characterisation of the secreted cytokines and the intracellular NfrA enabled a comparison of plasmid and chromosomal expression in two different contexts. Although the menadione reductase activity levels detected from chromosomal strains are lower than in other already investigated NTR-expressing strains (such as NmeNTR), the advantage of clinically relevant CP-506 prodrug for CDEPT approach might be of a bigger value (52).

We sought to quantify the production and secretion of the cytokines biochemically and to determine functional activity in cell culture assays. Furthermore, the effect of bacterial culture duration (as a function of growth phase) on cytokine production was assessed. For both cytokines, ELISAs indicated that plasmid-borne expression cassettes produced substantially higher levels of product in culture supernatants. By sampling cultures at multiple time points, we were able to track secretion of the cytokines across growth phases (**Figure 3B**). In previous studies, supernatants were analysed at a single time point (7-hour), which represents late-exponential phase growth in *C. sporogenes* (51). However, in the present study we sampled at three timepoints: 5, 7, and 10 hours, representing mid-exponential, late-exponential, and stationary growth phases. Regardless of the product or genetic construct, the highest product titres occurred at the 5-hour time point and were lower at 7 hours, decreasing further by 10 hours. An ELISA relies on target-specific antibodies and can detect the presence of correctly folded protein in its native conformation. However, it is not a direct measurement of protein function. We reasoned that testing the functionality of the recombinant cytokines in cell culture assays would yield more meaningful results. Proliferation of the cytotoxic T cell line CTLL-2, and the myeloid progenitor cell line FDCP-1, were used to quantify mIL-2 and mGM-CSF, respectively, in culture supernatants. These assays revealed a similar trend: plasmid-based constructs produced higher levels of proliferation and peak titres occurred at the earliest timepoints, diminishing over culture time.

Increased expression of plasmid-encoded products compared to those that are chromosomally integrated is widely reported in the literature (71). This is attributed to the presence of multiple copies of the plasmid in each cell, which enables a higher rate of transcription. However, the cause of the substantial variation in product titre between time points, for plasmid- and chromosome-based products of both cytokines, was unclear. We sought to investigate the cause of the decline in cytokine levels at later time points. We rationalised that there were two major factors that could influence yield: production and degradation. Some bacterial gene promoters have been shown to vary according to growth phase, such as that of the *ptb* gene of *Clostridium acetobutylicum* (72). For the promoter in question (P*fdx*) we did not find equivalent reports of growth-phase dependent expression.

We turned our attention to potential causes of degradation. The genome sequence of *C. sporogenes* reveals several coding sequences with homology to characterised *B. subtilis* protease genes, including several matrix metalloproteases and bacillolysin, all of which are secreted (73). Protease secretion from *C. sporogenes* occurs in the late exponential phase and is repressed by glucose, ammonia, phosphate, ATP/ADP and certain amino acids, suggesting protease secretion occurs when energy sources are depleted. Previous studies have speculated that it is the proteolytic capability of *C. sporogenes* that enables it to effectively colonise solid tumours (74, 75). Several studies have demonstrated that genetic manipulation of secreted proteases to increase or decrease proteolysis has been demonstrated in several studies, including in *B. subtilis* (76, 77) and *L. lactis* (78). We hypothesised that co-secretion of native proteases was adversely affecting the stability of our recombinant product, which would explain the reduction of cytokine activity at later time points. To test this hypothesis, we identified and deleted an operon of six genes in the *C. sporogenes* NCIMB 10696 genome related to proteolysis. Following deletion of this operon in the cytokine-expressing strains, the quantitative and functional assays were repeated. Increased levels of cytokine were observed in all samples but was most pronounced in samples from later time points, corresponding to late exponential and stationary phases, growth stages in which protease expression and secretion increases due to nutrient depletion.

Through the creation of an effective CRISPR-Cas9 system, this study illustrates the potential of recombinant *C. sporogenes* strains as drug delivery vehicles. As our collective knowledge of the biology and genetics of clostridia increases, alongside the creation of effective genetic tools that are genuinely accessible, our ability to fully exploit these species in pursuit of medical applications will be realised. While the results of this study are promising, they only demonstrate efficacy in the simple, contrived setup of *in vitro* cell assays. The logical next step is to determine efficacy *in vivo*, that is, the therapeutic efficacy of *Clostridium*-mediated immunotherapy in comparison to current standard practice. Selection of potent, “next generation” agents and high-throughput experiments to further optimise expression and secretion will increases the chance of success. However, trying to gauge efficacy by comparison to systemically administered agents may be futile. Perhaps it is time to leap into the unknown.

## Data availability statement

The original contributions presented in the study are included in the article/**Supplementary Material**, further inquiries can be directed to the corresponding authors. Plasmids pTetR-P*_IPL12_*-Cas9 and p8222F-g7-SLS-GGL will be made available through Addgene following publication. 

## Ethics statement

Ethical approval was not required for the studies on animals in accordance with the local legislation and institutional requirements because only commercially available established cell lines were used.

## Author contributions

AK, LC, YZ, and TB co-designed the study. AK, LC, YZ, and TB performed the experiments and collected and analysed the data. AK, TB, and KK wrote the manuscript. KK assisted in the conceptualisation and evolution of ideas. AK and TB initiated the project and share senior authorship. TB wrote the funding grant. All authors contributed to data interpretation, discussions, and revision of the manuscript. All authors contributed to the article and approved the submitted version.
