# Supplementary material for: Refined control of CRISPR-Cas9 gene editing in Clostridium sporogenes: the creation of recombinant strains for therapeutic applications
Source: Front Immunol. 2023 Oct 5;14:1241632. doi: 10.3389/fimmu.2023.1241632 (PMC10585264; doi:10.3389/fimmu.2023.1241632)
Supplement: Supplementary file 1 [file DataSheet_1.docx]

Supplementary Material

**Refined control of CRISPR-Cas9 gene editing in *Clostridium sporogenes*: the creation of recombinant strains for therapeutic applications**

Aleksandra M. Kubiak^1,2^, Luuk Claessen^1,2^, Yanchao Zhang^2^, Khashayarsha Khazaie^3*,^ Tom S. Bailey^2*^

*** Correspondence:** [Khazaie@mayo.ed](mailto:Khazaie@mayo.ed); [t.bailey@maastrichtuniversity.nl](mailto:t.bailey@maastrichtuniversity.nl)

**1. Supplementary materials**

## Construction of two vectors with anhydrotetracycline-inducible promoters and GusA reporter

Codon optimised TetR and two inducible promoters (P*IPL12* and P*fetO*) were synthesised by Integrated DNA Technologies (IDT) (Table S3). *gusA* gene was cloned from plasmid pRPF185 (1). Fragments were cloned by PCR and *BsaI* restriction sites were integrated at the termini of each fragment by addition of non-annealing sequences at the 5’ of primers. GusA expression plasmids bearing promoter P*IPL12* or P*fetO* were assembled into pGG212 by GGA, resulting in plasmids pGG212-P*IPL12* and pGG212-P*fetO*. The original pRPF185 vector, bearing the original inducible tetracycline promoter and *gusA* gene, was included in the study as a control. All three vectors were conjugated into *C. sporogenes*-NT strain.

## Validation of tetracycline-inducible systems by glucuronidase assay

The three *C. sporogenes*-NT strains containing a tetracycline-inducible GusA expression plasmid were grown overnight and then sub-cultured into fresh media at a 1:100 dilution. When the OD_600_ reached approximately 0.5, they were divided into three equal cultures. Two of them were induced with 32 ng/ml and 96 ng/ml of anhydrotetracycline, while the third one was left uninduced. After 4 hours, 1 ml of each culture was pelleted, and the GUS activity was evaluated as previously described (1, 2).

# Supplementary Figures

**Supplementary Figure S1**

**Figure S1: Schematic representation of the variable components of the p8222F knock-in vectors.** The upper panel shows the *SLS* operon-targeting vector (p8222F-g7-SLS-GGL), and the lower panel shows the *pyrE*-targeting vector (p8222F-g2-PyrE-GGL). gRNA numbers (gX) are arbitrary numbers based on the users panel of gRNA spacer sequences, typically determined using a validated algorithm such as CRISPOR. In both examples, the “GGL” suffix denotes a previously reported cloning site that is compatible with golden gate cloning and blue-white screening on Xgal (3).

**Supplementary Figure S2**

**Figure S2: Validation of tetracycline-inducible systems by glucuronidase assay in in *C. sporogenes*-NT.** Three *C. sporogenes*-NT strains containing anhydrotetracycline inducible, plasmid based GusA (section 1.1) were subjected to activity assay. GusA was placed under the control of P*tetO* in pRPF185 (1) or one of two modified P*fdx* promoters: P*IPL12* for pGG212-P*IPL12* and P*fetO* for pGG212-P*fetO.* Two inducer concentrations were tested (32 and 96 ng/ml), and compared to samples that were not induced. Data represent the mean ± s.d. of three biological replicates.

**Supplementary Figure S3**

**Figure S3: Gene editing at four different genome loci in *C. sporogenes*.** **(A)** gRNA and repair cassette sequences to target four separate genome loci were cloned into p8222F-gX-HC. Using the workflow described in Figure 1, four knockout mutants were created. Screening plate colonies by PCR following induction indicated that the target sequence was deleted in all clones. Sequences of the primers used in these screens are provided in Table S2. For the WT controls, a larger band is produced for *pyrE*, *spo0A* and *spoIIE*, indicating that deletion has not occurred. In the case of the SLS operon deletion, the PCR reaction failed for the WT control due to the large size of the locus (10,469 bp). L: denotes 1-kb Plus DNA Ladder (NEB). **(B)** Details of the deleted genome loci are provided, including gRNA spacer/protospacer sequences and calculated gene editing efficiency.

**Supplementary Figure S4**

**Figure S4: PCR analysis of five different cargo integrations in *C. sporogenes* strains
(*C. sporogenes* WT or *C. sporogenes*-Δ*pyrE)* performed using the two-plasmid CRISPR-Cas9 system targeting the *SLS* operon.** **(A)** Gel electrophoresis showing PCR screens confirming simultaneous deletion of *SLS* operon and integration of chosen cargo (NfrA, mIL-2, mGM-CSF, 3 kb λDNA and 5 kb λDNA). Randomly selected colonies (c1-c8/c10) were subjected to PCR reactions using chromosome-specific primers flanking the *SLS* locus (Table S2). L: denotes 1-kb Plus DNA Ladder (NEB), (1): *C. sporogenes-*NT control (1,844 bp) and (2) *C. sporogenes* WT (10,469 bp, failed PCR reaction due large PCR product and required lengthy extension time). **(B)** Summary of calculated genome editing efficiencies recorded for the eight integrant strains, based on the number of the total integrant colonies screened.

**Supplementary Figure S5**

**Figure S5: Visual confirmation of uracil auxotrophy.** Six integrant strains (harbouring NfrA, mIL-2 and mGM-CSF), in two different backgrounds (+/- *pyrE* gene) were streaked on defined medium plates (4), with and without supplementation of uracil (20 μl/ml). Plates were incubated under anaerobic conditions for 48h, and visual observations were recorded. Two strains: *C. sporogenes*-NT and *C. sporogenes*-NT-Δ*pyrE* were used as controls.

# Supplementary Tables

**TABLE S1: Plasmids and strains used in the study**

| **Plasmids** | **Relevant characteristics** | **Source** |
| --- | --- | --- |
| pMTL83151 | *E*. *coli*-clostridia shuttle vector | (5) |
| pGG212L | *E*. *coli*-clostridia shuttle vector: Golden Gate assembling vector based on pMTL82121, Cm^R^, Gram^-^ replicon p15a, Gram^+^ replicon pBP1 | (3) |
| pGG212-mIL-2 and pGG212-mGM-CSF | *E*. *coli*-clostridia shuttle vector harbouring codon optimised murine IL-2 / murine GM-CSF, driven by P*fdx* promoter (CLSPO_c0087) and nprM3 precursor (CLSPO_c14710) | This study,  Table S3 |
| pGG212-NfrA | *E*. *coli*-clostridia shuttle vector harbouring codon optimized B. subtilis NfrA nitroreductase (Uniprot P39605) driven by modified thiolase promoter (P*thl14)* | This study,  Table S3 |
| pTetR-P*_IPL12_*-Cas9 | *E*. *coli*-clostridia pMTL83151 shuttle vector with codon optimized *tetR* gene, driven by miniP*4*_tU promoter and *cas9* gene, controlled with inducible P*IPL12* promoter | This study,  Table S3 |
| p8222F | *E*. *coli*-clostridia shuttle vector (pMTL82221), Erm^R^, Gram^-^ replicon p15a, Gram^+^ replicon pBP1 containing inducible P*fetO* promoter | This study,  Table S3 |
| p8222F-g7 | p8222F with sgRNA number 7 targeting *SLS* operon | This study |
| p8222F-g7-SLS | p8222F-g7 with SLS repair template | This study |
| p8222F-g1-spo0A | p8222F with sgRNA number 1 and *spo0A* repair template | This study |
| p8222F-g2-spoIIE | p8222F with sgRNA number 2 and *spoIIE* repair template | This study |
| p8222F-g2-pyrE | p8222F with sgRNA number 2 and *pyrE* repair template | This study |
| p8222F-g7-SLS-3kb | p8222F-g7-SLS with 3kb Lambda DNA cargo | This study |
| p8222F-g7-SLS-GGL | p8222F-g7-SLS with the addition of the previously reported GGA cloning site between the homology arms (ISCIENCE-D-22-05626R3 accepted manuscript) | This study |
| p8222F-g2-PyrE-GGL | p8222F-g2-pyrE with the addition of the previously reported GGA cloning site between the homology arms (ISCIENCE-D-22-05626R3 accepted manuscript) | This study |
| p8222F-g7-SLS-mIL-2 | p8222F-g7-SLS with P*fdx*-nprM3-mIL-2 cargo | This study |
| p8222F-g7-SLS-mGM-CSF | p8222F-g7-SLS with P*fdx*-nprM3-mGM-CSF cargo | This study |
| p8222F-g7-SLS-NfrA | p8222F-g7-SLS with P*thl14*-NfrA cargo | This study |
| p8222F-g3-PR1 | p8222F sgRNA number 3 targeting metalloprotease operon | This study |
| **Strain** | **Description** | **Source** |
| *E. coli* 10-beta | Expression/ plasmid storage strain (Δ(ara-leu) 7697 araD139 fhuA ΔlacX74 galK16 galE15 e14- ϕ80dlacZΔM15 recA1 relA1 endA1 nupG rpsL (StrR) rph spoT1 Δ(mrr-hsdRMS-mcrBC)) | NEB^®^ C3019 |
| *E. coli* S17-1 | Conjugative donor strain (recA pro hsdR RP4­2­Tc::Mu­Km::Tn7 integrated into the chromosome) | ATCC 47055™ |
| *S. pyogenes /* Spy-20565 | *Streptococcus pyogenes* DSM20565: group A wild type strain | DSMZ Culture collection (Germany) |
| *C. sporogenes-*WT/ CspWT | *Clostridium sporogenes* NCIMB 10696 wild type strain | NCIMB culture collection (UK) |
| *C. sporogenes-*NT/ CspNT | *Clostridium sporogenes* Δ*SLS::BM1 - SLS* operon KO strain with inserted BM1 bookmark | (6) and this study |
| CspNT-pGG212-mIL-2 /  CspNT-pGG212-mGM-CSF | *C. sporogenes* *SLS* KO strain secreting plasmid-based murine IL2 / murine GM-CSF cytokine, placed under control of P*fdx* promoter and nprM3 signal sequence | (6) and this study |
| CspNT-pGG212-NfrA | *C. sporogenes* *SLS* KO strain secreting plasmid-based NfrA nitroreductase placed under control of P*thl14* promoter | This study |
| CspWT-pTetR-P*_IPL12_*-Cas9 | *C. sporogenes* WT strain harbouring codon optimized TetR driven by driven by miniP*4*_tU promoter and *cas9* gene, controlled with inducible P*IPL12* promoter | This study |
| CspWTΔ*spo0A* | *C. sporogenes* WT strain with deleted *spo0A* gene (CLSPO_c18650) | This study |
| CspWTΔ*pyrE* | *C. sporogenes* WT strain with deleted *pyrE* gene (CLSPO_c33380) | This study |
| CspWTΔ*spoIIE* | *C. sporogenes* WT strain with deleted *spoIIE* gene (CLSPO_c37040) | This study |
| CspNTΔ*pyrE* / CspNTΔP | *C. sporogenes*-NT *SLS* KO strain with deleted *pyrE* gene | This study |
| CspNT::3kb | *C. sporogenes*-NT strain with inserted 3kb λDNA fragment at *SLS* locus | This study |
| CspNT::5kb | *C. sporogenes*-NT strain with inserted 5kb λDNA fragment at *SLS* locus | This study |
| CspNT::mIL-2 /  CspNT::mGM-CSF / CspNT::NfrA | *C. sporogenes*-NT strains with inserted murine IL-2 / murine GM-CSF / NfrA nitroreductase gene at *SLS* locus (placed under control of P*fdx* promoter and *nprM3* secretion peptide for cytokines and P*thl14* for the NTR) | This study |
| CspNTΔP::mIL-2 /  CspNTΔP::mGM-CSF / CspNTΔP::NfrA | *C. sporogenes* NT strain with deleted *pyrE* gene and inserted murine IL-2 / murine GM-CSF gene at *SLS* locus (placed under control of P*fdx* promoter and *nprM3* secretion peptide for cytokines and P*thl14* for the NTR) | This study |
| CspNTΔPΔPR1 | *C. sporogenes* NT *SLS* KO strain with deleted *pyrE* gene and *nprM* proteolytic operon | This study |
| CspNTΔPΔPR1::mIL-2 / CspNTΔPΔPR1::mGM-CSF /  CspNTΔPΔPR1::NfrA | *C. sporogenes* NT strain with deleted *pyrE* gene and *nprM* proteolytic operon, harbouring murine IL-2 / murine GM-CSF gene at *SLS* locus (placed under control of P*fdx* promoter and *nprM3* secretion peptide for cytokines and P*thl14* for the NTR) | This study |
|  |  |  |

**TABLE S2: List of oligonucleotides used in the study**

| **Primer name** | **Sequence (5’-3’)** | **Description** |
| --- | --- | --- |
| P*fdx*-*Bsa*IGG-F | TACGGGTCTCGTCCAGTGTAGTAGCCTGCGAAATAAGTAAGG | Amplification of promoter-signal sequence module for mIL-2/mGM-CSF |
| nprM3-*Bsa*I-R | CCGGTCTCAGAGCAGCATAAACTGCAGAAACAGTAGAAAGAGTG |  |
| mIL2-*Bsa*I-F | CCGGTCTCAGCTCCAACTTCTAGTTCAACTTCAAG | Amplification of mIL-2 |
| mIL2-*Bsa*I-R | TATAGGTCTCTGTCTTTATTGAGGACTTGTAGATATGATACTTTGACAG |  |
| GMCSF-*Bsa*I-F | CCGGTCTCAATGCTGCTCCAACAAGAAGTCCTATAACTGTAAC | Amplification of mGM-CSF |
| GMCSF-*Bsa*I-R | TATAGGTCTCTGTCTTTATTTCTGACCAGGTTTCTTACATTCAAAAGG |  |
| P*thl14*-*Bsa*I-F | TACGGGTCTCGTCCAGCATAAGTTTAATTTTTTTGTTAAAAAATATTAAAC | Amplification of P*thl14* promoter for NfrA |
| P*thl14*-*Bsa*I-R | CCGGTCTCATCATATATATAAACCTCCTTTATTTTATTACGTTTTCTC |  |
| NfrA1-*Bsa*I-F | CCGGTCTCAATGAACAATACAATAGAAACTATATTAAACCAC | Amplification of NfrA gene |
| NfrA1-*Bsa*I-R | TATAGGTCTCTGTCTTTAGTTCTTATTGAATCCTTTTTCTTTTACATAATCATTTAG |  |
| tyrS-BB-*Bsa*I-F | CCGGTCTCACACGAAGGGACGATTGATTATGGCGCGCCGCCATTATTTTTTTGAACAATTGAC | Amplification of pMTL83151 backbone with partial *tyrS* and *pepN* terminators |
| pepN-BB-*Bsa*I-R | CCGGTCTCAACCTCTAAAAGGTGATTTTTATTTATAAATTACCTGCACGATCGGTCTTGCCTTGCTCGTC |  |
| pepN-BB-*Bsa*I-F | CCGGTCTCAAGGTGGTTTTTTTATTTATAAATTACTGTATCACCATGATTACGAATTCGCGGCCGCTCAGTCACCTCCTAGCTG | Amplification of Cas9 fr. 1 |
| Cas9Δ*BsmB*Ip1 | CCGGTCTCAAGCCAAGAAGAATTTTATAAATTTATCAAACCAA |  |
| Cas9Δ*BsmB*Ip2 | CCGGTCTCAGGCTTGCTCCCCCATCAATATAACC | Amplification of Cas9 fr. 2 |
| Cas9-*Bsa*I-F | CCGGTCTCTATGGATAAGAAATACTCAATAGGCTTAGATATCGGC |  |
| P*IPL12*-TetR-*Bsa*I | CCGGTCTCTCCATATATAACACACCTCCTTAAAAATTACACAAC | Amplification of inducible promoter module and TetR |
| tyrS-*Bsa*I-R | CCGGTCTCACGTGTAAACGAAGGGGCGTTTTTTATTTCAGGAAACAGCTATGACCTTAAGACCCACTTTCACAT |  |
| sgRNA-UNI-R | CCGACGTCATAAAAATAAGAAGCCTGCAAATGCAGGCTTCTTATTTTTATAAAAAAAGCACCGACTCGGTGCCACTTTTTCAAGTTG | Guide RNA 3’and terminator, for construction of complete guide RNAs in primer dimer reaction. |
| *Sal*I-SG2-pyrE-F | TAGTCGACATGTATAGTTGATAGAAGAGGTTTTAGAGCTAGAAATAGCAAGTTAAAATAAGGCTAGTCCGTTATCAACTTGAAAAAGTGGCACCGAGTCGGTGCTTTTTTTATAAAAATA | Creation of guide number 2 retargeting sequence for *pyrE* in combination with sgRNA-UNI-R |
| pyrE-RHA-*Aat*II-F | TATAGACGTCCCTAAAGAAGGAAATAATGGCATAAGAAT | Amplification of left and right homology arm to generate the editing template to delete *pyrE* |
| pyrE-RHA-*Bsa*I-R | CCGGTCTCATTAAATAATTCCCCTTATTTCTTCTAAAGTTTGAATAC |  |
| pyrE-LHA-*Bsa*I-F | CCGGTCTCATTAAATTTAAAAATAAGGAGTGTCTCAAAATAGATTTAA |  |
| pyrE-LHA-*Asc*I-R | TATAGGCGCGCCAGTTGTTCCAGATGTTGATGTATACTGCTTATTTG |  |
| pyrE-Chr-FII | GGATACAAACAAGCAGAAGACATTCCTATTTC | Colony PCR chromosomal screening primers to confirm Δ*pyrE* KO |
| pyrE-Chr-RII | GTGGTATAGGAGCATGTCTTGTATGTACTTG |  |
| *Sal*I-SG1-*spo0A*-F | TAGTCGACGTACAACTCCTTTGTCACTGGTTTTAGAGCTAGAAATAGCAAGTTAAAATAAGGCTAGTCCGTTATCAACTTGAAAAAGTGGCACCGAGTCGGTGCTTTTTTTATAAAAATA | Creation of guide number 2 retargeting sequence for *pyrE* in combination with sgRNA-UNI-R |
| Spo0A-LHA-*Aat*II-F | CCGACGTCGAAATTGTGAAGGAAAAGACTTAAAAATAGTGGCTTATAG | Amplification of left and right homology arm to generate the editing template to delete s*po0A* |
| Spo0A-LHA-*Bsa*I-R | CCGGTCTCAGGTCTTAATTATTATCTAATATTCCAGCATCCTTTAGCATC |  |
| Spo0A-RHA-*Bsa*I-TAG-ZsG1-F | CCGGTCTCAGACCATGAAGTACCGCATGGAGGTATGGATATATTAACAGAGTTTATATTTTTAGGTCTACGGG |  |
| Spo0A-RHA-*Asc*I-R | TATAGGCGCGCCCCAATCTTTACTATCTTTTTTTACATCTGCTCTGTCATACATC |  |
| spo0A-Chr-R | CCTGCAGCTGTATCCGGTATACAAATAGGAGATAG | Colony PCR chromosomal screening primers to confirm Δ*spo0A* KO |
| spo0A-Chr-F2 | GGTCATTTTTAGGTTTATTCCCGTAGACC |  |
| SalI-SG2-*spoIIE*-F | GGCCGTCGACTTAATTATAGGATATGTAAAGTTTTAGAGCTAGAAATAGCAAGTTAAAATAAGGCTAGT | Creation of guide number 2 retargeting sequence for *spoIIE* in combination with sgRNA-UNI-R |
| spoIIE-LHA-*Aat*II-F | TATAGACGTCGACTTAAAATCCTGCGGGCCTAACAG | Amplification of left and right homology arm to generate the editing template to delete spoIIE |
| spoIIE-LHA-*Bsa*I-R | CCGGTCTCATATTAATAATTAATCACCCCCATTAAGTTACTTCTC |  |
| spoIIE-RHA-*Bsa*I-F | CCGGTCTCAAATATAGAAAGAGAATATAAAGTTAAGTAGTAAATAC |  |
| spoIIE-RHA-*Asc*I-R | TATAGGCGCGCCCTATCCTAGGATTTAGTTCATATTTTTCAC |  |
| spoIIE-Chr-R | CTCTCTTATCTATTATTACCCTTTGTGTT | Colony PCR chromosomal screening primers to confirm Δ*spoIIE* KO |
| spoIIE-Chr-F | ATATAATTATTTATGTCGTCAGCAATGAGAC |  |
| *Sal*I-SG7-SLS-F | CGGTCGACGCAGTACCTGTACTAAAGGAGTTTTAGAGCTAGAAATAGCAAGTTAAAATAAGGCTAGTCCG | Creation of guide number 7 retargeting sequence for *SLS* operon in combination with sgRNA-UNI-R |
| SLS-LHA-*Aat*II-F | GACGTCGGAAAATAAAGAAGGACAAGTATTGCTAAATTCTTATGATGTAG | Amplification of left and right homology arm to generate the editing template to delete *SLS* |
| SLS-LHA-*Bsa*I-R | CCGGTCTCAGACGCCTCAAATTTATTATACCTAATAATCC |  |
| SLS-RHA-*Bsa*I-F | CCGGTCTCAGACGCCTTAGTATAGTAATTTTATTTGCAG |  |
| SLS-RHA-*Asc*I-R | GGCGCGCCCCAAAGTTCACTAATTTTAACTTTAACAAGAAACCTG |  |
| SLS-Chr-F | TAGCATCAGGAGGAACGAAGATAAAGGC | Colony PCR chromosomal screening primers to confirm *SLS* KO |
| SLS-Chr-R | CCATAAATCTCTCAATATGTCAAAGCCATCAAGTCC |  |
| SLS-LHA- *BsmB*Ic-R | TCCGTCTCATCCACCTCAAATTTATTATACCTAATAATCCT | Amplification of *SLS* LHA and RHA introducing *BsmB*I to ligate with GG compatible LacZ fragment |
| SLS-RHA- *BsmB*Ic-F | GAACGTCTCGCTGACCTTAGTATAGTAATTTTATTTGCAGTAAG |  |
| LacZ-*BsmB*I-F | TGGATGAGACGGAGGATAATCAATCGTCCCTTC | Amplification of GG-LacZα fragment |
| LacZ-*BsmB*I-R | TCAGCGAGACGTTCAGAACAGCGCGCCA |  |
| pyrE-LHA- *BsmB*Ic-R | TCCGTCTCATCCATTAAATAATTCCCCTTATTTCTTCTAAAGTTTGAATAC | Amplification of *pyrE* LHA and RHA introducing *BsmB*I to ligate with GG compatible LacZ fragment |
| pyrE-RHA- *BsmB*Ic-F | GAACGTCTCGCTGAATTTAAAAATAAGGAGTGTCTCAAAATAGATTTAATTTTG |  |
| 3kb-*BsmB*I-F | CGTCTCCTGGAGACCGCGACTTACCATGTATCTCGTG | Amplification of 3 kb and 5 kb λDNA fragments |
| 3/5kb-*BsmB*I-R | CGTCTCTTCAGCAGATATTGCTGCAACGGTCGATTGCC |  |
| 5kb-*BsmB*I-F | CGTCTCCTGGAGTTGAATGGCATGGTCGCTGGC |  |
| Loss-traJ-F | GGATAGGTGAAGTAGGCCCACC | Control PCR amplifying traJ, to confirm loss of plasmids in edited strain |
| Loss-traJ-R | GTCATGGCTCTGCCCTCGG |  |
| *Sal*I-SG3-PR1-F | TAGTCGACGTTGATAAAGAGCATGATTGGTTTTAGAGCTAGAAATAGCAAGTTAAAATAAGGCTAGTC | Creation of guide number 3 retargeting sequence for nprM operon in combination with sgRNA-UNI-R |
| PR1-*Aat*II-LHA-F | TATGACGTCGGATATGCAAGTTTTGGAAGAC | Amplification of left and right homology arm to generate the editing template to delete *nprM* proteolytic operon |
| PR1-*Bsa*I-LHA-R | CCGGTCTCGAAGGGTATATAGGTATATTTATATAGAG |  |
| PR1-*BsaI*-RHA-F | CCGGTCTCACCTTCAATTTTTAAAATTCAGTATATTATATGTGAA |  |
| PR1-*Asc*I-RHA-R | ACGGCGCGCCGCCCCGTTCGTAAGAAAAATAG |  |
| PR1-Chr-F2  (AK324) | GAAGTTATGGAAGGCTACGAAAAGGAAAG | Colony PCR chromosomal screening primers to confirm ΔPR1 proteolytic KO |
| PR1-Chr-R2 (AK325) | GGTGGAGGTTTTAATCACAGATTTGGA |  |
| PR-Int (AK323) | CCCAAGATATTGTTGTGGTTTTCGGT |  |

**TABLE S3: DNA fragments and codon-optimized genes synthesized by IDT** (where applicable: restriction enzyme recognition sites underlined)

| **Fragments** | **5’-3’ sequence** |
| --- | --- |
| P*IPL12*-P*miniP4_Ut* | GGTCTCTCCATATATAACACACCTCCTTAAAAATTACACAACTTTATACGATGTTCATTATAA**CCTATCAATGATAGA**AGCTCAATACTTATAATTTTTGACAAATTTATTTTTTAAAGTTAAAATTAAGTTGTTAGAGAAAACGTATAAATTAGGGATAAACTATGGAACTTATGAAATAGATTGAAATGGTTTATCTGTTACCCCGTATCAAAATTTAGGAGGTTAGTTAGA |
| TetR_CO | ATGTCAAGATTAGATAAAAGTAAAGTAATTAACTCAGCATTAGAGTTACTTAATGAGGTAGGAATAGAAGGTTTAACAACCAGAAAATTAGCTCAGAAGTTGGGTGTAGAGCAGCCTACATTGTATTGGCATGTAAAAAATAAGAGAGCTTTATTGGATGCCTTAGCTATTGAGATGTTAGATAGACATCATACTCACTTTTGCCCTTTAGAAGGGGAAAGCTGGCAAGATTTTTTAAGAAATAACGCTAAAAGTTTTAGATGTGCTTTACTTAGTCATAGGGATGGAGCAAAAGTACATTTAGGTACAAGACCTACAGAAAAACAGTATGAAACTTTAGAAAATCAATTAGCCTTTTTATGCCAACAAGGTTTTTCACTAGAGAATGCATTATATGCATTAAGCGCTGTGGGGCATTTTACTTTAGGTTGCGTATTGGAAGATCAAGAGCATCAAGTTGCTAAAGAAGAAAGGGAAACACCTACTACTGATAGTATGCCACCATTATTAAGGCAAGCTATAGAATTATTTGATCACCAAGGTGCAGAGCCAGCATTTTTATTCGGTCTTGAATTAATAATTTGCGGATTAGAAAAACAACTTAAATGTGAAAGTGGGTCTTAA |
| P*fetO* | CCTCTAGAAAATTACTTTAAAA**TCTATCATTGATAGG**GTAAAATATAAATCGGTCGACTTAGCATTTTGCATAGACGTCTTGCATTAATCGTTAGGCGCGCCGTTC |
| P*fdx*-nprM3 | GGTCTCGTCCAGTGTAGTAGCCTGcGAAATAAGTAAGGAAAAAAAAGAAGTAAGTGTTATATATGATGATTATTTTGTAGATGTAGATAGGATAATAGAATCCATAGAAAATATAGGTTATACAGTTATATAAAAATTACTTTAAAAATTAATAAAAACATGGTAAAATATAAATCGTATAAAGTTGTGTAATTTTTAAGGAGGTGTGTTACATATGAAAAGTAAAAAATTATTAGCTACAGTGCTAAGTGCTGTAATCACTCTTTCTACTGTTTCTGCAGTTTATGCTgctctgagacc |
| P*thl14* | GCATATGCATAAGTTTAATTTTTTTGTTAAAAAATATTAAACTTTGTGTTTTTTTTAACAAAATATATTGATAAAAATAATAATAGTGGGTATAATTAAGTTGTTAGAGAAAACGTAATAAAATAAAGGAGGTTTATATAT |
| NfrA | ATGAACAATACAATAGAAACTATATTAAACCACAGAAGTATAAGGTCTTTCACAGATCAACTTTTAACTGCTGAAGAGATTGATACTTTAGTTAAAAGTGCACAAGCTGCATCTACATCAAGTTATGTACAGGCTTATTCAATTATAGGTGTAAGTGATCCAGAAAAGAAAAGAGAACTTTCTGTTTTAGCAGGAAATCAACCATACGTTGAGAAAAATGGACATTTCTTTGTATTTTGTGCTGACCTATATAGACATCAGCAATTAGCAGAAGAGAAGGGTGAACATATTAGTGAATTATTGGAAAATACAGAGATGTTTATGGTAAGTCTAATTGATGCCGCATTAGCTGCACAAAATATGAGTATAGCTGCTGAATCTATGGGTTTAGGAATATGTTATATTGGTGGTATTAGGAATGAACTAGATAAGGTAACCGAAGTTTTACAAACTCCTGATCATGTATTACCTTTATTTGGATTAGCTGTTGGTCATCCAGCAAACTTAAGTGGAAAAAAGCCTAGATTACCAAAACAAGCTGTATACCATGAAAATACATACAATGTAAACACTGACGATTTTAGGCATACAATGAACACTTATGATAAGACTATAAGTGATTATTATAGAGAAAGAACAAACGGTAAGCGTGAAGAAACATGGTCTGATCAAATATTAAATTTTATGAAACAGAAACCTAGAACATATCTAAATGATTATGTAAAAGAAAAAGGATTCAATAAGAAC |
| mIL-2 | GCTCCAACTTCTAGTTCAACTTCAAGTTCTACAGCTGAAGCACAACAACAGCAACAACAGCAACAACAACAGCAACAACATCTTGAACAACTATTAATGGATCTACAAGAACTTCTATCTAGAATGGAAAATTATAGAAACCTTAAACTACCAAGAATGCTAACATTTAAATTTTATTTACCAAAACAAGCAACAGAATTAAAAGATCTTCAGTGTCTCGAAGATGAACTTGGTCCTCTACGTCATGTTCTAGATTTAACTCAAAGTAAAAGTTTTCAATTGGAAGATGCAGAAAATTTTATAAGTAATATTAGAGTAACTGTTGTAAAACTAAAGGGATCTGATAACACTTTTGAATGTCAATTCGATGATGAATCAGCTACTGTTGTAGATTTTCTAAGAAGGTGGATAGCATTCTGTCAAAGTATCATATCTACAAGTCCTCAATAA |
| mGM-CSF | GCTCCAACAAGAAGTCCTATAACTGTAACAAGACCATGGAAACATGTTGAAGCAATTAAAGAAGCTCTTAATTTATTAGATGACATGCCTGTAACTTTAAATGAAGAGGTTGAAGTTGTGTCTAACGAATTTTCATTTAAAAAGTTAACATGTGTTCAAACTAGGTTAAAGATATTCGAGCAAGGACTTAGAGGTAATTTTACTAAATTGAAAGGAGCATTAAATATGACCGCTTCTTATTATCAAACATACTGTCCACCTACTCCAGAAACCGATTGCGAAACTCAAGTAACAACTTATGCAGATTTTATAGATTCTCTAAAAACATTTTTAACAGATATTCCTTTTGAATGTAAGAAACCTGGTCAGAAATAA |
| GGL | TGGATGAGACGGAGGATAATCAATCGTCCCTTCGTGTAAACGAAGGGGCGTTTTTTATTTCAGGAAACAGCTATGACCGCGGCCGCTGTATCCATGAGACCAAAGCGGGCAGTGAGCGCAACGCAATTAATGTGAGTTAGCTCACTCATTAGGCACCCCAGGCTTTACACTTTATGCTTCCGGCTCGTATGTTGTGTGGAATTGTGAGCGGATAACAATTTCACACAGGAAACAGCTATGACCATGATTACGCCAAGCTTGCATGCCTGCCGGTCCACGCTAGAGGATCCCCGGGTACCGAGCTCGAATTCATTAGCTGTTGTGTTGCAACGTCGTGACTGGGAAAACCCTGGCGTTACCCAACTTAATCGCCTTGCAGCACATCCCCCTTTCGCCAGCTGGCGTAATAGCGAAGAGGCCCGCACCGATCGCCCTTCCCAACAGTTGCGCAGCCTGAATGGCGAATGGCGCCTGATGCGGTATTTTCTCCTTACGCATCTGTGCGGTATTTCACACCGCATTTGGTGCACTCTCAGTACAATCTGCTCTGATGCCGCATAAGGTCTCAAGACATGCAAGCTTGGCACTGGCCGTCGTTTTACACTAGCATAAAAATAAGAAGCCTGCATTTGCAGGCTTCTTATTTTTATGGCGCGCTGTTCTGAACGTCTCGCTGA |

**Supplementary Materials: References**

1. Fagan RP, Fairweather NF. *Clostridium difficile* has two parallel and essential Sec secretion systems. J Biol Chem. 2011;286(31):27483-93.

2. Mordaka PM, Heap JT. Stringency of Synthetic Promoter sequences in *Clostridium* revealed and circumvented by tuning promoter library mutation rates. ACS Synthetic Biology. 2018.

3. Bailey TS, Hittmeyer P, Zhang Y, Kubiak AM. Streamlined assembly of cloning and genome editing vectors for genus *Clostridium*. iScience. 2023:107484.

4. Lovitt RW, Morris JG, Kell DB. The growth and nutrition of *Clostridium sporogenes* NCIB 8053 in defined media. J Appl Bacteriol. 1987;62(1):71-80.

5. Heap JT, Pennington OJ, Cartman ST, Minton NP. A modular system for *Clostridium* shuttle plasmids. Journal of Microbiological Methods. 2009;78:79-85.

6. Kubiak AM, Bailey TS, Dubois LJ, Theys J, Lambin P. Efficient secretion of murine IL-2 from an attenuated strain of *Clostridium sporogenes*, a novel delivery vehicle for cancer immunotherapy. Front Microbiol. 2021;12:669488.
